# Assessing Orthorexic Tendencies and Dietary Patterns: A Cross-Sectional Study of Orthorexia Nervosa and Dietary Patterns in Lithuania

**DOI:** 10.3390/nu18040616

**Published:** 2026-02-13

**Authors:** Rron Lecaj, Inga Iždonaitė-Medžiūnienė, Olga Kavoliūnienė, Aleksandra Batuchina

**Affiliations:** SMK College of Applied Sciences, Liepų, LT-92195 Klaipėda, Lithuania

**Keywords:** eating behavior, orthorexia nervosa, food frequency, dietary pattern, eating disorder

## Abstract

**Background/Objectives:** Orthorexia nervosa (ON) is an emerging condition marked by a preoccupation with healthy eating that is linked to diminished well-being and social functioning. While research on ON extends across countries, no studies about ON have been found in Lithuania. This study aimed to investigate dietary patterns, socio-demographic correlates, and the prevalence of orthorexic tendencies in a Lithuanian adult sample. **Methods:** A cross-sectional online survey was conducted using the ORTO-R 6-item scale, a Food Frequency Questionnaire (FFQ), and socio-demographic and dietary behavior measures. Principal component analysis (PCA) was applied to the FFQ to identify dietary patterns, and stepwise multiple linear regression was used to examine predictors of orthorexic tendencies. **Results:** Approximately 15% of the Lithuanian adult sample exhibited elevated orthorexic tendencies, while three dietary factors were extracted, including Balanced-Traditional, Processed-Dense and Protein-Rich patterns. Both Balanced-Traditional and Protein-Rich dietary patterns were positively associated with orthorexic tendencies, although only the Balanced-Traditional pattern remained a significant predictor in the fully adjusted regression model, which explained 16.2% of the variance in ORTO-R scores (F(7,468) = 12.97, *p* < 0.001). Higher orthorexic tendencies were associated with following a dietary plan, adherence to the Healthy-Traditional pattern, being female, younger age, higher meal frequency, employment status, and being married. **Conclusions:** Orthorexic behaviors were more prevalent among younger women, individuals following structured diets, and those adhering to health-oriented eating patterns. These findings highlight the interplay between demographic and dietary factors in shaping orthorexic tendencies in the Lithuanian population.

## 1. Introduction

Orthorexia nervosa (ON) is a condition which was initially coined in European research in Italy [1]. The condition appears to impair social functioning as it is often linked to poorer mental health outcomes [2]. Across Europe and Asia while using different ON instruments, researchers have consistently confirmed its unequivocal prevalence and presence [3,4]. Within the European context, Eastern European countries seem to have assimilated Western ideals of beauty and eating behavior [5]. However, research in the Baltic countries including Latvia, Lithuania, and Estonia is scarce. Particularly for Lithuania, research on eating behaviors relates to pediatric and adolescent populations, with some studies revealing emotional eating patterns among students with higher stress levels [6,7,8,9]. Therefore, it is important to understand eating behaviors and styles across the Lithuanian population above 18 years old. Furthermore, it is important to understand the emergence of orthorexic tendencies in Lithuania to add to the body of literature on ON.

### 1.1. Orthorexia Nervosa

From its initial conception, ON or correct eating has been regarded as a maladaptive behavior borrowed from Western values and consumerism ideology [10]. Prior to ON, healthism was the unofficial term used non-scientifically [11,12,13]. Since then, ON emerged—scientifically and empirically—following the need for a label related to the Diagnostic and Statistical Manual (DSM-V) [14]. Trends on research related to ON portray a linear emergence of the condition in health, nutrition, and mental health research across Europe, with spikes in the early 2000s, 2010s and 2020s following COVID-19 [1,15].

Scoping further, European researchers have collaborated on consensus to ensure that ON becomes an official annex and be labeled as a disorder within the DSM. The symptoms outlined suggest that ON is a Feeding and Eating Disorder whereby individuals possess a preoccupation with rigid eating patterns which affect psychological, social, and economic functioning [2]. Furthermore, the diagnosis consensus argues ON’s distinction from Anorexia Nervosa (AN) and obsessive–compulsive disorder (OCD) owing to ON-specific properties such as compulsions being related to nutrition only, and the preoccupation with nutritious correct eating irrelated to body image [2]. Moreover, a series of factors have been linked to ON across countries, including time spent on social media and social media addictive behaviors, excessive physical exercise and gym culture, and emotion dysregulation and educational/professional background [2].

Difficulties in identifying, recognizing and regulation emotions has been found in several samples, including Lebanon, Germany, Turkey and digitally [16,17,18,19]. Individuals with ON tendencies employ dysfunctional metacognitive processes wherein they have difficulties reflecting and monitoring their own thinking processes mediated by adaptive or maladaptive emotional regulation abilities [19]. Such a finding is in line with studies on perfectionism and eating disorders wherein individuals diagnosed with an eating disorder (ED) scored higher on perfectionism and dysfunctional metacognitive beliefs, worry and rumination [20]. To that end, profiles of high-ON-scoring individuals include high scores for alexithymia, emotion regulation difficulties, body dissatisfaction, and excessive physical exercise [21].

Excessive exercise has also been a predominant risk factor for ON [2]. Exercise addiction has been associated with high self-expectations and higher tendencies for ON [22]. Other studies also have found higher ON prevalence across aerobic and strength-training exercise levels, internal exercise motivation and other measures [22,23,24]. Rather, the condition has been more tightly linked to muscle dysphoria and excessive exercise in CrossFit populations mediated by social media content online and tendencies for perfectionism [25]. Following that, social media addiction, use and type of content have also been linked to higher ON tendencies [25,26]. Platforms like Reddit, Instagram and TikTok have exacerbated ON symptoms among populations that set high self-expectations, exercise, and tend to compulsively obsess over nutritious content in food [27,28]. Out of these platforms, however, Instagram has become a recovery space for self-diagnosed ON individuals [3].

Nevertheless, the work toward better defining ON and understanding its properties remains a long journey. The lack of an official and APA-approved diagnostic body influences researchers, particularly related to measuring symptomatology [29]. The meta-analytic review evaluating the reliability of various ON measuring instruments, however, has not halted the research process. A majority of researchers have been using the ORTO-R, the Dusseldorf Orthorexia Scale (DOS) and its translations, and the Orthorexia Nervosa Inventory (ONI) [25]. Fewer studies have utilized the Eating Habits Questionnaire (EAQ-21) [15,25]. The scarcity of these instruments leads to a lack of comparable data, further extending the issue of receiving an APA-approved diagnostic model for ON.

### 1.2. Eating Patterns Across Europe

Emerging patterns of ON prevalence and overall eating behavior across European contexts highlights the need for a response from dietitians and mental health practitioners alike [2]. As a result of the World Health Organization’s (WHO) and relevant agencies’ efforts toward lowering obesity and chronic metabolic illnesses, eating behavior has shifted significantly in the past decade across European countries [30]. For example, a cross-national analysis of 22 European countries found that in European adults, 14 to 44% of their total energy intake comes from ultra-processed foods [31]. To track specific dietary behaviors, Food Frequency Questionnaires (FFQs) have been validated across contexts and languages, including Germany, France, Sweden, Italy and Poland [32,33,34,35,36]. Cross-checking through FFQ-based literature, there are cultural similarities and Westernization effects occurring in terms of dietary patterns. German adults consume diets rich in refined grains, processed meats and added fats [33]. This is similar to the UK, the Netherlands and Belgium wherein a consumption of ultra-processed food (UPF) is present [31]. France shows a more moderate consumption of UPF regardless of its western European position, and adults in the country are more prone to consuming stable meat-heavy diets or present mixed dietary patterns [32]. A significant shift is underlined in southern European countries where adults from Italy, Spain and Portugal consume fewer UPFs and more vegetables, legumes and olive oil [31,35]. Northern European countries, including the Scandinavian region, exhibit unique eating behavior patterns where high caffeine consumption and moderate intake of dairy and sweets is noted [34,37].

These studies also highlight significant differences in demographic predictors of specific dietary patterns, suggesting that those with higher socioeconomic status (SES) tend to consume more fruits, vegetables, and legumes in comparison to their lower SES counterparts [37]. Furthermore, age seems to be a fine predictor as adults in Italy, as well as younger adults, rely on packaged snacks and ready-made meals, which emphasizes the Westernization effect [35].

### 1.3. The Case of Lithuania

While research on eating behavior patterns of adults in Lithuania is scarce, dietary pattern analyses using FFQs in Lithuania consistently identify a diet characterized by frequent consumption of cereals and cereal products, root vegetables, legumes, fruits, vegetables, and fish. In a nationally representative study of Lithuanian adults, a dominant dietary pattern was labeled the cereals pattern, which loaded positively on bread and cereal products, potatoes, vegetables, fruits, and legumes and was distinct from patterns characterized by meat or energy-dense foods [38]. This pattern reflects staple foods traditionally consumed in Lithuania and aligns with long-standing culinary practices shaped by seasonality, local agriculture, and food storage requirements. Subsequent FFQ-based studies confirm the persistence of this plant-forward dietary component within Lithuanian populations. Longitudinal analyses indicate gradual increases in vegetable consumption and modest shifts toward more diversified plant food intake, while cereals and root vegetables remain core components of daily diets [38,39]. Research among Lithuanian young adults similarly identify dietary patterns marked by higher consumption of vegetables, fruits, grains, and fish, contrasting with protein-dominant and highly processed dietary patterns [9]. In addition, contemporary data from national food consumption studies of Lithuanian adults show that Lithuanians tend to consume about 1691 kilocalories (kcal) per day with many micronutrient insufficiencies and maintain a consistent calorie deficit [40]. Lithuania presents a landscape where dietary patterns are heavily predicted by demographic factors such as age (young adults adhere less to Mediterranean dietary patterns) and education (higher education levels were strongly associated with healthier food consumption patterns) [9,40,41]. Yet there are no studies measuring the prevalence of ON in Lithuania. One study reported that young adults, women, and individuals actively seeking clean or healthy food were more likely to submit to dietary supplements, making up 78.1% of the entire study’s sample [42]. Such trends in different European countries and in Lithuania present the need to measure ON in an effort to develop prevention, intervention, and postvention measures in relation to significant predictors.

Utilizing the Revised ORTO-15 scale (ORTO-R) and an adaptation of the Food Frequency Questionnaire (FFQ) to the Lithuanian context, a representative sample of 1091 adults of various socio-demographic backgrounds were surveyed online. The aim of the study was to assess the prevalence of ON in a Lithuanian sample and to identify profiles of vulnerable groups and their dietary patterns. This study is one of the first of its kind for Lithuania. It adds to the current body of literature and informs relevant stakeholders, including nutrition and mental health practitioners and relevant national and international organizations (the WHO, etc.) related to the current situation and needs.

## 2. Materials and Methods

### 2.1. Study Design and Participants

This cross-sectional study was conducted among adults living in Lithuania in an attempt to assess ON tendencies and determine how these tendencies associate with eating behaviors and food consumption patterns. A total of 1091 participants completed the questionnaire and were included in the analysis; the criteria for inclusion ensured the participation of adults older than 18 years old residing in Lithuania. Given the exploratory nature of the study, the sample size was determined pragmatically, aiming to maximize participation and ensure stable multivariate analyses rather than to test a predefined hypothesis. This explains the gender imbalance present in the study, owing to which caution is warranted in making gender-related conclusions. The data were collected through a random sampling technique via a self-administered survey combining different instruments from March to June 2025. A Google Forms questionnaire was administered. The study was conducted in compliance with the 1964 Declaration of Helsinki’s ethical standards. The research protocol was reviewed and approved by the Research Ethics Committee of SMK College of Applied Sciences. All participants were informed about the purpose, procedures, and voluntary nature of the research before taking part. Informed consent was obtained from all respondents prior to survey completion. Confidentiality and anonymity of the participants’ responses were strictly maintained throughout the research process.

### 2.2. Measures

#### 2.2.1. Orthorexia Tendencies

The ORTO-R is a self-report questionnaire composed of 6 items evaluated on a 1–5 Likert scale where 1 = never, 2 = rarely, 3 = sometimes, 4 = very often, and 5 = always [43]. The questionnaire was forward translated to Lithuanian and back translated to English by the 4-person research team to ensure internal consistency, and it was piloted on a sample of 30 initial participants based on validation principles of 5 participants per item [44]. Higher total scores on the ORTO-R reflect higher levels of orthorexic tendencies. In the current sample, the scale demonstrated acceptable internal consistency (Cronbach’s α = 0.78), with corrected item–total correlations ranging from 0.47 to 0.63.

#### 2.2.2. Food Frequency Questionnaire (FFQ)

Dietary patterns were assessed using a 17-item food frequency questionnaire adapted for Lithuanian adults. Participants indicated how often they consumed major food groups (e.g., cereals, vegetables, fruits, meats, dairy, fats, sweets, soft drinks, alcohol, and coffee) on a Likert-type scale where 0 = never/seldom (less than once a month), 1 = 1–3 times per month, 2 = 1–2 times per week, 3 = 3–4 times per week, and 7 = daily consumption. The FFQ showed good internal consistency in the present study (Cronbach’s α = 0.84). Corrected item–total correlations ranged from 0.28 to 0.60, and no item substantially decreased scale reliability, supporting the cohesiveness of the measure for evaluating dietary habits.

#### 2.2.3. Socio-Demographic and Lifestyle Variables

In an attempt to identify and compile profiles of ON-vulnerable individuals, several socio-demographic and lifestyle variables were assessed. Socio-demographic variables included age, gender, economic household income, household size (persons residing), height, weight, employment status, and marital status. Furthermore, lifestyles were measured by questions on diet behavior, diet source, type of diet, and reasons for dieting.

#### 2.2.4. Statistical Analysis

All statistical analyses were performed using IBM SPSS Statistics, Version 21.0 (IBM Corp., Armonk, NY, USA). Prior to analysis, all variables were screened for missing data, outliers, normality, and appropriate coding. Descriptive statistics (means, standard deviations, frequencies, and percentages) were computed for all socio-demographic, dietary, and ORTO-R variables. To identify underlying dietary patterns, a principal component analysis (PCA) with Varimax rotation was applied to the Food Frequency Questionnaire (FFQ). Sampling adequacy was evaluated using the Kaiser–Meyer–Olkin (KMO) statistic and Bartlett’s test of sphericity. Components with eigenvalues greater than 1.0 and clear interpretability were retained. Regression-based factor scores for each extracted component were generated and used as continuous variables in subsequent analyses. Bivariate associations between orthorexic tendencies (ORTO-R mean score), dietary patterns, and socio-demographic variables were examined using Pearson’s correlation coefficients. Assumptions of linearity and homoscedasticity were evaluated through scatterplots and standardized residual diagnostics. To determine the strongest predictors of orthorexic tendencies, a stepwise multiple linear regression model was conducted with the mean ORTO-R score as the dependent variable. Independent variables included age, gender, marital status, employment status, household size, daily meal frequency, current diet plan status, and the three FFQ-derived dietary factor scores. Entry and removal criteria followed SPSS defaults (*p* < 0.05 for entry; *p* > 0.10 for removal). Model fit was evaluated using R^2^, adjusted R^2^, and F-statistics. Multicollinearity was assessed with Tolerance and Variance Inflation Factor (VIF) values, with VIF < 2 indicating acceptable levels. The Durbin–Watson statistic was used to verify the independence of residuals. Statistical significance for all analyses was set at *p* < 0.05 (two-tailed).

## 3. Results

### 3.1. Participant Characteristics and ON Prevalence

The majority of the participants were women (76.4%), with fewer participants reporting as men (22.9%) or preferring not to say their gender (0.6%), whereas the mean age was 33 years (M = ±33.06). Women had a mean age of 33.29 years (SD = 12.24), while men had a mean age of 32.40 years (SD = 12.52). Body mass index (BMI), calculated as weight in kilograms divided by height in meters squared, had a mean value of 24.91 kg/m^2^ (SD = 4.95). As shown in Table 1, the majority of our participants reported living with 3–4 household members (65.6%). Most participants reported being married (41.8%) or in stable relationships (33.6%), while 17.8% were single and 5.7% were divorced. Participants were also asked to describe how many times they eat full meals per day (excluding snacking), to which almost half of the participants (48%) responded by saying they eat two full meals per day, whereas fewer (32.2%) reported three meals, 15.7% reported one meal, and 4.2% reported more than three meals per day. Their dietary behaviors varied as almost half of the participants (40.6%) reported being on some type of diet.

Considering the nature of ORTO-R where 1 = never and 5 = always, the mean calculated for the Lithuanian sample (M = 2.73; SD = 0.83) indicates moderate levels of orthorexic tendencies across our sample (see Table 2). In the absence of validated diagnostic cutoff scores for the ORTO-R, a distribution-based approach was applied. Participants scoring at or above the 85th percentile of the sample distribution (M ≥ 3.67) were descriptively categorized as exhibiting relatively elevated orthorexic tendencies. Based on this criterion, approximately 15% of the sample met this threshold exhibiting elevated orthorexic tendencies. Importantly, this percentage represents an estimation of individuals at higher risk for orthorexic behaviors based on the distribution of ORTO-R scores and should not be interpreted as a clinical diagnosis or a national prevalence estimate.

### 3.2. Dietary Patterns

Principal component analysis of the Food Frequency Questionnaire was conducted to identify underlying dietary patterns. Sampling adequacy was confirmed (KMO = 0.86), and Bartlett’s test of sphericity was significant (χ^2^(136) = 4546.24, *p* < 0.001). Three dietary components were retained based on eigenvalues greater than 1 and scree plot inspection, accounting for a cumulative value of 49.6% of the total variance. The extracted patterns were labeled Balanced-Traditional Diet, Protein-Rich Diet, and Processed-Dense Diet. Regression-based standardized factor scores were computed and retained for subsequent analyses. The *Balanced-Traditional Diet* was labeled based on high loadings of water, fresh fruits and vegetables, root vegetables, cereals, dairy products, meat, fish, and fats, reflecting a pattern consistent with culturally rooted, minimally processed, and nutritionally balanced eating practices and compliant with Lithuanian culinary practices. The *Protein-Rich Diet* was defined by strong loadings of fish and seafood, nuts and seeds, legumes, and eggs, indicating a dietary pattern centered on protein-dense foods commonly associated with health-oriented and structured eating behaviors. In contrast, the *Processed-Dense Diet* was characterized by high consumption of soft drinks and juices, savory snacks, alcoholic beverages, and sugar-rich foods, representing a pattern dominated by energy-dense, highly processed, and discretionary food items. Importantly, the PCA presented in Table 3 captures patterns of food consumption rather than prescriptive nutritional guidelines. The FFQ was constructed to assess the frequency of broad food groups without a priori classification based on macronutrient or micronutrient composition, allowing dietary patterns to emerge empirically from observed eating behaviors.

### 3.3. Correlations

To examine the correlations occurring between variables, Person’s correlation analyses were conducted. As depicted in Table 4, Pearson’s correlation analyses indicated a significant positive association between ORTO-R scores and both the Healthy-Traditional dietary pattern (r = 0.216, *p* < 0.001) and the Protein-Rich dietary pattern (r = 0.230, *p* < 0.001). No significant association was observed between ORTO-R scores and the Processed-Dense dietary pattern (r = −0.006, *p* = 0.855).

### 3.4. Stepwise Regression Analysis

Owing to the large set of exploratory predictors for the current study, a stepwise linear regression analysis was chosen to identify the dietary behavior and socio-demographic variables that were most strongly correlated with orthorexic tendencies. Such an analytic method allows for the identification of combinations that uniquely contribute to orthorexic tendencies in the absence of a theoretical framework [45]. Results are interpreted cautiously given known limitations of stepwise procedures, including sensitivity to variable entry order and potential model instability.

In the current study, the total ORTO-R score was calculated as the dependent variable, while predictors (including dietary behavior and socio-demographic variables) were automatically input stepwise. All assumptions of multiple regression were evaluated, including linearity, homoscedasticity, normality of residuals, multicollinearity and influential cases. Tolerance and VIF values were within acceptable limits, indicating no problematic multicollinearity.

In Model 1, the strongest single predictor was whether people followed a dietary plan (coded 1 = yes; 2 = no). This explained the largest proportion of explained variance. Model 2 introduced Healthy/Traditional dietary pattern scores followed by gender in Model 3 (1 = female; 2 = male). Models 4 and 5 introduced marital status and number of meals per day, and Model 6 added employment status. Once Model 7 introduced age (see Table 5), all variables were shown to significantly improve model fit, as indicated by the change in F values across steps. The final model explained 16.2% of the variance in orthorexic tendencies across the Lithuanian sample (adjusted R^2^ = 0.150).

While the model explains a relatively small percentage of the variance, it still accounts for a meaningful proportion of the overall sample. Given the multifactorial nature of orthorexia and the cross-sectional design, a substantial amount of variance remains unaccounted for, likely reflecting psychological constructs (e.g., perfectionism, anxiety, rigidity, and health beliefs) not measured in this dataset. In the fully adjusted model, several variables were significantly associated with higher ON scores.

Following a dietary plan emerged as the strongest predictor of orthorexic tendencies in the final model (β = −0.211, *p* < 0.001), indicating that individuals actively adhering to a structured diet reported greater orthorexic tendencies; the negative coefficient reflects the reverse coding of this variable. Greater adherence to the Healthy-Traditional dietary pattern also predicted higher orthorexic tendencies (β = 0.167, *p* < 0.001). Gender was significant, with women scoring higher than men (β = −0.135, *p* = 0.001), reflecting the coding of the variable. Marital status was similarly associated with orthorexic tendencies (β = 0.130, *p* = 0.001), with partnered individuals reporting slightly higher scores than single participants. Eating a greater number of full meals per day predicted higher orthorexic tendencies (β = 0.131, *p* = 0.009), as did being employed (β = 0.122, *p* = 0.008). Age showed a negative association (β = −0.103, *p* = 0.023), indicating that younger participants exhibited higher orthorexic tendencies. While several predictors reached statistical significance, effect sizes were in the small-to-moderate range (see Table 6). Given the cross-sectional design, these findings reflect associations rather than causal or temporal relationships.

### 3.5. Summary of Findings

Overall, participants exhibit moderate levels of orthorexic tendencies, and three dietary patterns are identified (Balanced-Traditional, Protein-Rich, and Processed-Dense). These three dietary patterns accounted for over half of the variance within the given sample. In addition, correlates emerge, as those adhering to the Balanced-Traditional and Protein-Rich dietary patterns tend to have higher orthorexic tendencies than those adhering to the Processed-Dense dietary pattern. Following that, the stepwise regression analysis indicates that orthorexic tendencies were associated and predicted by individuals’ adherence to a dietary plan close to the Balanced-Traditional dietary pattern. Women, individuals in relationships, employed participants, younger participants and those consuming more meals per day reported higher orthorexic tendencies. While the nature of this study is exploratory, these findings provide key indications regarding dietary behavior and eating patterns that correlate with higher orthorexic tendencies in the general population in Lithuania.

## 4. Discussion

This study aimed to examine the current dietary landscape and tendencies for ON in a general population sample in Lithuania through a cross-sectional design. Combining tools such as the FFQ and ORTO-R and the analyses conducted within the study make this the first of its kind within the Lithuanian context. In this exploratory study, the use of PCA, Pearson’s correlation and stepwise linear regression analyses enabled us to define current dietary patterns in Lithuania, assess ON prevalence and correlates, and identify dietary patterns most significantly associated with ON.

The present findings demonstrate significant associations between dietary behaviors, dietary patterns, and selected socio-demographic characteristics in relation to orthorexic tendencies. This study represents the first population-based investigation in Lithuania to assess both the prevalence of orthorexic tendencies and food frequency-derived dietary patterns in a general adult sample. Behavioral variables reflecting structured eating practices, including adherence to a dietary plan, consumption of a greater number of full meals per day, and employment status, were associated with higher orthorexic tendency scores. In addition, orthorexic tendencies were positively associated with health-oriented dietary patterns, specifically the Balanced-Traditional and Protein-Rich patterns, whereas no association was observed with the Processed-Dense dietary pattern. Altogether, these findings indicate that orthorexic tendencies in this Lithuanian sample were more closely related to structured, normatively valued eating behaviors and health-focused dietary choices rather than to patterns characterized by higher consumption of processed foods. Furthermore, two of the dietary patterns of this study’s FFQ, namely Balanced-Traditional and Protein-Rich diets, portray an array of food groups that are socially acceptable and perceived as healthy in comparison to the Processed-Dense food groups. Additionally, these food groups may be preferentially chosen by individuals with higher orthorexic tendencies due to their perceived healthfulness. Still, this study raises the question of whether some dietary patterns signal broader eating psychopathology, while others may be more characteristic of orthorexic tendencies. This ties into existing evidence which shows that ON is closely related to higher health consciousness, dietary control and socially reinforced norms as healthy and “clean” as well as functional and “optimal” eating rather than pathological eating alone [46,47]. Furthermore, previous research also highlights that a vegan diet did not directly result in disordered eating, but the prevalence of ON was higher in vegans and vegetarians in comparison to people consuming meat [48]. Vegetarian or vegan diets could increase the risk of ON and individuals’ perception of “healthy” eating promoting thinness and weight loss, with the idea that ON encounters motivation associated with weight and/or body shape [49,50]. A similar study in student populations in Poland combined the FFQ and ORTO-15, revealing that students reluctantly eating meat products were less likely to score high on ON tendencies [51]. While the current study found that Protein-Rich diets among Lithuanians scored higher on the ORTO-R, this indicates that consuming protein-rich foods such as meat may reveal a broader relationship with higher ON tendencies. Protein consumption has recently grown in popularity among the broader population, and specifically among athletes, as media promotes protein as a muscle builder while also promoting muscularity as an ideal bodily trait [52,53]. This finding connects to several studies related to ON which highlight that CrossFit, gym-goers, and athletes generally exhibit higher orthorexic tendencies [22,54,55]. Nevertheless, a deeper investigation of the interaction of gender identity, sex, and type of exercise/physical activity alongside chronotypes/sleep quality and other symptomatology is warranted.

The gender-specific orientation of ON is further confirmed as the current study also revealed a higher prevalence of ON among women, although this may be, in part, due to the imbalance of the sample distribution. Findings of one study highlighted a higher prevalence of ON symptoms among women, and the association between ON and a specific interest toward dietary choices [55]. A multitude of studies on ON across cultural contexts show that women are more likely to develop ON, with only few studies indicating this trend in men [24,25,54,55]. Other studies also argue that ON may show up differently in men than in women, with men prioritizing protein consumption over healthy eating with a link to muscularity ideation [52,55]. However, the current study did not examine exercise behavior, muscularity ideation or men under the ON lens. Furthermore, being younger in this study was associated with higher tendencies for ON. One study even stated that younger participants admitted having increased habits of healthy eating that became pathological and resulted in malnutrition [55]. Several other studies revealed a decline in orthorexic tendencies as age increased in their samples, attributing more rigid dietary patterns to younger populations [24,56]. The current study confirms this string of findings for the context of Lithuania. Yet some of the socio-demographics considered in this study remain underexplored in the literature. While our findings indicate that being in a relationship and being employed can be significant correlates and predictors of ON, other studies seldom consider employment and relationship status to be significant [56,57]. Being employed may be connected to higher ON scoring since full-time employment may enforce routines and healthy lifestyles for people [57]. Similarly, being in a relationship may be connected to partners sharing the same meals and, therefore, the same dietary patterns or beliefs about eating clean [24]. However, these variables remain to be explored by further research across cultural contexts and interrelation behaviors.

Importantly, these results reinforce the conceptualization of ON as a spectrum of disordered eating behaviors that overlap with, but remain distinct from, other eating disorders. The theoretical implications of this study focus on the alignment with previous findings that inadequate health-oriented eating may evolve into maladaptive eating patterns causing orthorexic risks. This study strengthens the conceptualization of ON as a part of a broader disordered eating background overlapping with other eating disorders but staying different at the same time. The study reveals that these eating patterns in Lithuania highlight the importance of cultural environment and regional dietary habits in orthorexia nervosa research, focusing on potential cross-cultural aspects of eating behavior and its triggers.

### 4.1. Implications

The findings of this study carry several practical and public health implications. First, the higher prevalence of orthorexic behavior among younger women and individuals with highly structured diets show the demand of targeted health education promoting balanced, rich and flexible eating without any assessment of food choices. Second, the study findings also presuppose the need for health professional and educator training to recognize the early stages of orthorexia nervosa risks, especially in populations that follow strict healthy diets and fall into the categories of younger age, women, being in a committed relationship, and being employed. Third, the study results demonstrate the reason for creating culturally based prevention diets in Lithuania as there is an increased interest in diet trends and wellness culture popularity. Fourth, the research may also propose various awareness campaigns and mindfulness development events in schools, universities, fitness centers or other environments where the risk of orthorexia nervosa prevalence tends to run high.

### 4.2. Limitations and Future Research

The research limitations include the methodological background (quantitative research), geographical location (Lithuania), and the research sample (which does not fully represent separate demographic groups such as older adults, teenagers, certain profession representatives, etc.). The practical application of orthorexia nervosa is also limited as this concept is too complex, clinically severe and remains non-clinically standardized. All the aspects discussed above make up a foundation for further research. Apart from application, several methodological limitations must be acknowledged. Participant recruitment via online dissemination resulted in a non-probabilistic sample with a marked overrepresentation of women, which may have influenced the observed associations, particularly given known gender differences in health-focused eating behaviors and orthorexic tendencies. In addition, the questionnaire was only administered digitally, which may have unintentionally excluded potential respondents owing to a lower level of digital literacy. Dietary patterns were derived from a FFQ that assessed consumption frequency but did not capture portion sizes, restricting the ability to estimate absolute intake or total dietary load. Additionally, a substantial proportion of participants reported currently following a dietary plan, reflecting contemporary dietary norms; however, the study design does not and cannot draw conclusions regarding whether dieting precedes, results from, or co-occurs with orthorexic tendencies. Consequently, ON is not a clinically standardized diagnosis, and although the ORTO-R shows improved psychometric performance compared to earlier instruments, the findings should be interpreted as reflecting orthorexic tendencies rather than clinical prevalence. Finally, the current study is missing several key socio-demographic factors and psychological symptoms that have been explored in concurrent research, including education level, social media behavior/use, perfectionism, and anxiety and depressive symptoms.

This study revealed the importance of exploring ON developmental trends and its potential transition into other eating disorders in the long term (longitudinal research). Future research would benefit more from some psychosocial correlations being highlighted (such as anxiety, perfectionism, social media influence, etc.). This will help researchers to project a more holistic profile of orthorexia nervosa risks. Additionally, seeking a deeper approach, such as qualitative or mixed-method study design, would help researchers reveal the lived experiences of individuals with orthorexic tendencies and would provide deeper insights into the phenomenon. Cross-cultural (cross-national) studies would contribute to determining whether the eating patterns observed in Lithuania reflect broader global trends (including various additional demographics) and may reveal how clearly orthorexia nervosa can be shaped by sociocultural factors.

## 5. Conclusions

This study provides population-wide insights into orthorexic tendencies and dietary patterns among Lithuanian adults across socio-demographic variables. Although ON is not formally recognized as a clinical diagnosis, it is increasingly examined in research as a pattern of maladaptive health-oriented eating behaviors. Accordingly, the present study focused on orthorexic tendencies rather than clinical diagnosis, using the ORTO-R as a validated instrument to assess behavioral patterns related to excessive preoccupation with healthy eating. As such, the findings reflect exploratory efforts of understanding dietary patterns and how they may relate to the specific behaviors connected to ON. The authors do not claim ON as a diagnosis, but rather as a pattern of behavior that may become maladaptive and dysfunctional, advising caution regarding Lithuanian dietitians and healthcare workers’ practices.

Furthermore, the findings link to a series of pan-European research efforts demonstrating the clinical and statistical prevalence of ON. Current findings indicate that orthorexic tendencies are more prevalent among women, young adults, employed individuals, and partnered individuals that are prone to adhering to dietary plans, such as the Balanced-Traditional and Protein-Rich dietary patterns identified in this study. The lack of an association between the Processed-Dense dietary pattern and orthorexic tendencies further demonstrates how ON fits into broader health-oriented eating behaviors. Overall, the results underscore the importance of examining cultural and contextual factors when examining orthorexic tendencies, as well as conducting further research on profiling individuals prone to ON while initiating campaigns and programs that increase awareness around clean, sustainable and flexible eating.

## Figures and Tables

**Table 1:** Participant characteristics.

Characteristic	Category	n (%)
Gender	Woman	834 (76.4%)
	Man	250 (22.9%)
	Prefer not to say	7 (0.6%)
BMI (kg/m^2^)	Mean ± SD	24.91 ± 4.95
Age (years)	Total/mean ± SD	33.06 ± 12.29
	Man/mean ± SD	33.29 ± 12.24
	Woman/mean ± SD	32.40 ± 12.52
Marital status	Single	187 (17.8%)
	Married	439 (41.8%)
	In a relationship	353 (33.6%)
	Divorced	60 (5.7%)
	Widowed	11 (1.0%)
Employment status	Student	217 (20.3%)
	Employed full-time	594 (55.7%)
	Employed part-time	96 (9.0%)
	Freelance	47 (4.4%)
	Unemployed	102 (9.6%)
	Other (shift work/parental leave)	11 (1.0%)
Household size	Living alone	140 (13.0%)
	2 persons	151 (14.0%)
	3–4 persons	708 (65.6%)
	≥5 persons	81 (7.4%)
Full meals per day	One	169 (15.7%)
	Two	516 (48.0%)
	Three	346 (32.2%)
	More than three	45 (4.2%)
Currently following a diet	Yes	442 (40.6%)
	No	648 (59.4%)

**Table 2:** ORTO-R mean.

Variable ^1^	M	SD	Min	Max
ORTO-R Total Score	2.73	0.83	1.00	5.00

^1^ Note. M = mean; SD = standard deviation. ORTO-R scores range from 1 (never) to 5 (always), with higher scores indicating greater orthorexic tendencies.

**Table 3:** Principal component analysis (PCA) of the Food Frequency Questionnaire (FFQ) items.

Food Group ^2^	Balanced-Traditional	Protein-Rich	Processed-Dense
Pure water	0.79		
Fresh vegetables	0.69		
Fresh fruits	0/64		
Root vegetables	0.58		
Cereals and cereal products	0.49		
Milk and dairy products	0.60		
Meat and meat products	0.64		
Fats (vegetable and animal)	0.46		
Fish and seafood		0.76	
Nuts and seeds		0.68	
Legumes		0.65	
Eggs		0.44	
Soft drinks and juices			0.74
Savory snacks			0.72
Alcoholic beverages			0.65
Sugar and sweets			0.59

^2^ Note. Principal component analysis with varimax rotation was conducted. Three components were retained based on eigenvalues > 1 and scree plot inspection, accounting for 49.6% of the total variance.

**Table 4:** Pearson correlations between ORTO-R scores and dietary patterns.

Variable ^1^	1	2	3	4
ORTO-R Total score		0.216 ***	0.230 ***	−0.006
Balanced-Traditional Diet			0.196 ***	0.251 ***
Protein-Rich Diet				0.011
Processed-Dense Diet				

^1^ Note. Values represent Pearson correlation coefficients. *** *p* < 0.001. N = 931 for correlations involving dietary patterns.

**Table 5:** Stepwise regression coefficients, ΔR^2^ by step, and model fit indices.

Model	Predictors Entered	R	R^2^	ΔR^2^	Adj. R^2^	SE	F Change	*p* (F Change)
1	Are you currently on a dietary plan?	0.215	0.046	0.046	0.044	0.8465	22.979	<0.001
2	+ Balanced-Traditional eating pattern (FFQ Factor 1)	0.305	0.093	0.047	0.089	0.8263	24.476	<0.001
3	+ Marital status	0.344	0.119	0.025	0.113	0.8155	13.572	<0.001
4	+ Gender	0.365	0.133	0.014	0.126	0.8096	7.993	0.005
5	+ Full meals per day	0.382	0.146	0.012	0.136	0.8046	6.804	0.009
6	+ Employment Status	0.391	0.153	0.008	0.142	0.8019	4.224	0.040
7	+ Age	0.403	0.162	0.009	0.150	0.7983	5.170	0.023

**Table 6:** Stepwise linear regression models predicting orthorexic tendencies.

Predictor	B ^1^	SE	β	*p*
Are you currently on a dietary plan?	−0.366	0.078	−0.211	<0.044
Balanced-Traditional Diet (FFQ Factor 1)	0.151	0.040	0.167	<0.001
Marital status	0.133	0.044	0.130	0.001
Gender	−0.268	0.086	−0.135	0.001
Full meals per day	0.131	0.050	0.131	0.009
Employment status	0.096	0.036	0.122	0.008
Age	−0.007	0.003	−0.103	0.023

^1^ Note. B = unstandardized coefficient; SE = standard error; β = standardized coefficient. ORTO-R = orthorexia nervosa tendencies score. N = 931.

## Data Availability

The data presented in this study are available upon request from the corresponding author (sharing of the data is contingent upon a third-party participator of the study, and the corresponding author must require permission upon due diligence of why the data is being requested).

## Abbreviations

The following abbreviations are used in this manuscript:

ON	Orthorexia Nervosa
ORTO-R	ORTO-15 Revised
FFQ	Food Frequency Questionnaire
AN	Anorexia Nervosa
WHO	World Health Organization
PCA	Principal Component Analysis
ED	Eating Disorders
APA	American Psychiatry Association
DSM	Diagnostic Statistical Manual
UPF	Ultra-Processed Food
